# Case Report: Five-Level Unilateral Laminectomy Bilateral Decompression (ULBD) by Two-Stage Unilateral Biportal Endoscopy (UBE)

**DOI:** 10.3389/fsurg.2022.944509

**Published:** 2022-07-05

**Authors:** Wen-long Wang, Zheng Liu, Si-jun Wu

**Affiliations:** Department of Orthopedics, Shougang Hospital, Health Science Centre, Peking University, Beijing, China

**Keywords:** case report, unilateral laminectomy bilateral decompression (ULBD), unilateral biportal endoscopy (UBE), lumbar spinal stenosis (LSS), multilevel

## Abstract

**Introduction:**

Unilateral biportal endoscopy (UBE) is a relatively new yet common minimally invasive procedure in spine surgery, capable of achieving adequate decompression for lumbar spinal stenosis through unilateral laminectomy bilateral decompression (ULBD). Neither additional fusion nor rigid fixation is required, as UBE-ULBD rarely causes iatrogenic lumbar instability. However, to our knowledge, five-level ULBD *via* two-stage UBE without lumbar fusion has been yet to be reported in the treatment of multilevel lumbar spinal stenosis.

**Case description:**

We present a case of an 80-year-old female patient who developed progressive paralysis of the lower extremities. Radiographic examinations showed multilevel degenerative lumbar spinal stenosis and extensive compression of the dural sac and nerve roots from L1-2 to L5-S1. The patient underwent five-level ULBD through two-stage UBE without lumbar fusion or fixation. One week after the final procedure, the patient could ambulate with walking aids and braces. Moreover, no back pain or limited lumbar motion was observed at the 6-month follow-up.

**Conclusion:**

Multilevel ULBD through UBE may provide elderly patients with an alternative, minimally invasive procedure for treating spinal stenosis. This procedure could be achieved by staging surgeries. In this case, we reported complaints of little back pain, despite not needing to perform lumbar fusion or fixation.

## Introduction

Unilateral biportal endoscopy (UBE) is a minimally invasive procedure in which water-medium endoscopic surgery is performed to achieve neural decompression or spinal fusion. Unilateral laminectomy bilateral decompression (ULBD) *via* UBE is indicated for lumbar spinal stenosis (LSS), as confirmed by several randomized controlled trials (1, 2). Although many studies on this technique have published, most only reported 1- or 2-level UBE-ULBD. This is the first report on 5-level ULBD *via* two-stage UBE for multilevel LSS.

## Case Description

**Figure 1:**
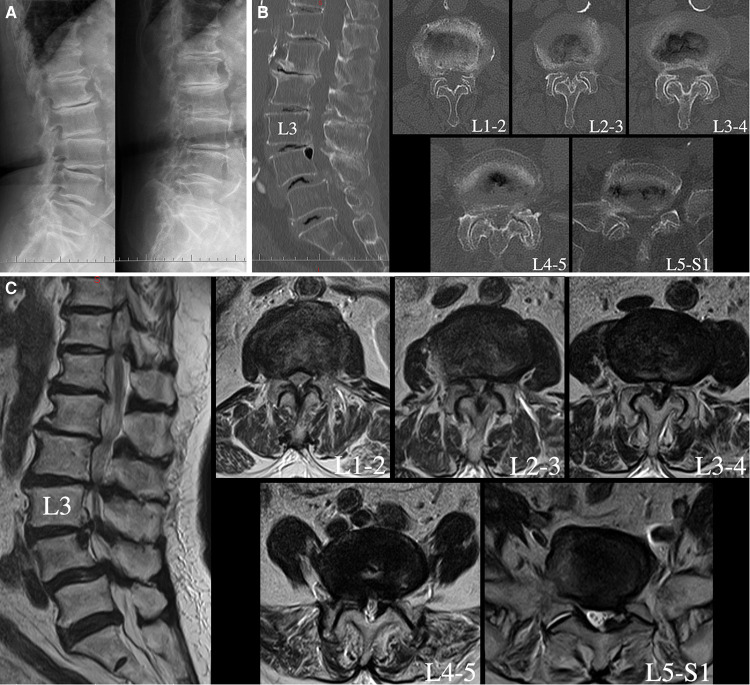
Flexion and extension lateral lumbar X-ray radiograph demonstrating relative dynamic stability at all lumbar segments (**A**). Lumbar CT showing hyperplasia and cohesion of the facet joints, ossification of the ligamentum flavum and multilevel stenosis of the lateral recesses and central canals (**B**). Sagittal and axial T2-weighted lumbar MRI revealing serious central canal stenosis from L1-2 to L4-5, lumbar disc herniation and left lateral recess stenosis at L5-S1 (**C**).

The patient was an 80-year-old female who developed progressive paralysis of the lower extremities and radicular pain of the left leg, which confined her to a wheelchair daily over the past two years. Sensory disturbances of the anterior bilateral thighs, lateral crura and dorsum of the feet were found through physical examination. The Lasegue sign of the left leg was positive, and the bilateral Babinski signs were negative. Flexion and extension lateral lumbar radiographs showed relative dynamic stability at all lumbar segments (Figure 1A). Lumbar computed tomography (CT) showed multilevel degenerative stenosis and L4 spondylolisthesis (Figure 1B). Magnetic resonance imaging (MRI) revealed serious stenosis from L1-2 to L5-S1 and left lateral recess stenosis at L5-S1 (Figure 1C).

**Figure 2:**
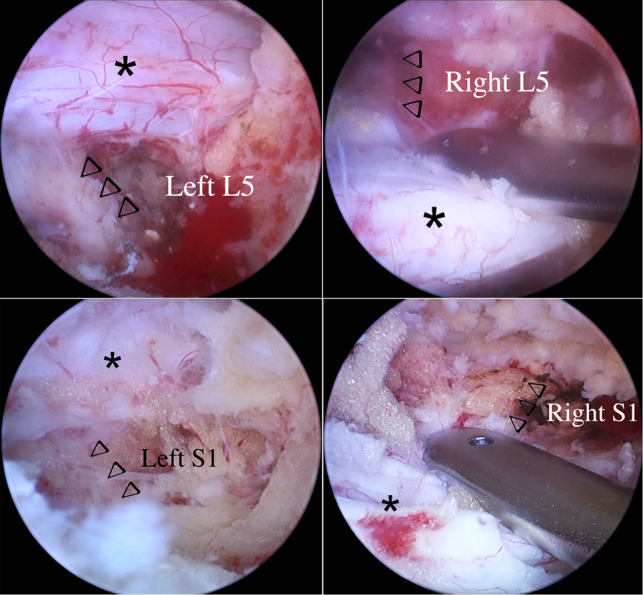
Bilateral L5 and S1 nerve roots and dural sacs after first-stage first stage UBE-ULBD (arrowheads indicate nerve roots, asterisks indicate dural sacs).

According to the patient's symptoms, physical examination, and imaging findings, the patient did not have significant lumbar instability, which was also the indication of non-fusion surgery. Because of multilevel severe stenosis and extensive neurological defects, it was difficult to identify one or two segments as the responsible segments. One stage surgery would take longer operation time and more intraoperative blood loss. So we performed staged procedures. In the first stage procedure, the patient's radicular pain of lower limb was considered, double ULBD at L4-5 and L5-S1 from the left side was performed through first-stage UBE to decompress the bilateral L5 and S1 nerve roots and dural sacs (Figure 2).

**Figure 3:**
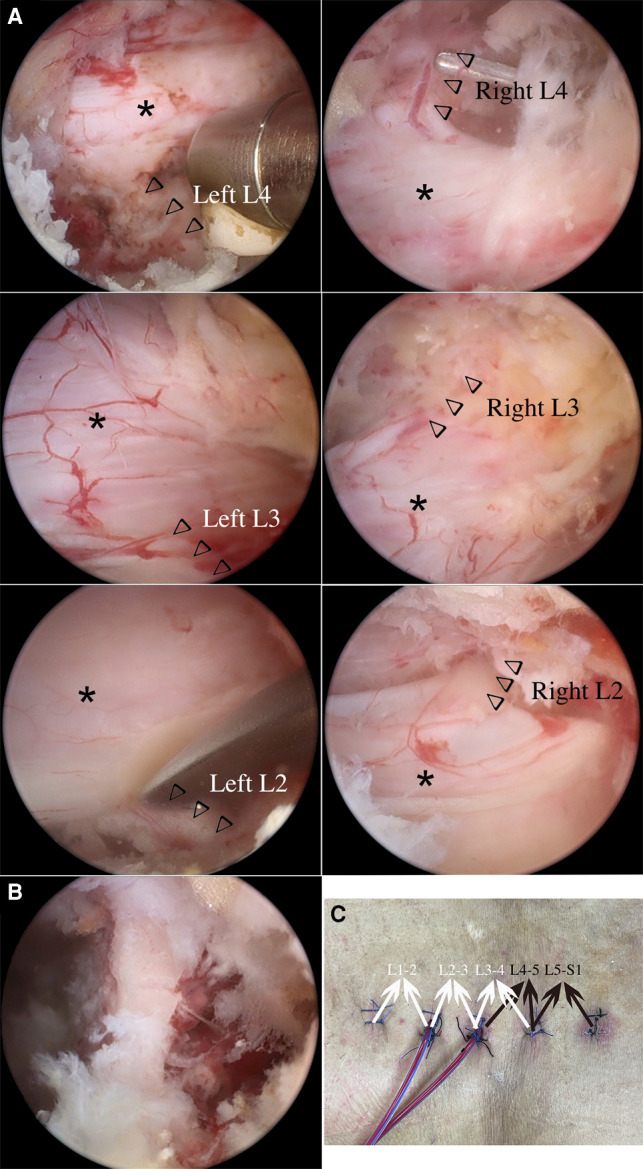
Bilateral L4, L3 and L2 nerve roots and dural sacs after second-stage UBE-ULBD (arrowheads indicate nerve roots, asterisks indicate dural sacs) (**A**). Residual left lamina of L4 after L4-5 and L3-4 UBE-ULBD (**B**). The surgical incisions after the two-stage procedure and the counterparts of the surgical segments (black arrowheads indicate the first-stage operation, white arrowheads indicate the second-stage operation) (**C**).

The left leg pain improved significantly the first day after the first-stage procedure. But the patient still could not realize to walk independently. So we continued to perform ULBD at L3-4, L2-3 and L1-2 from the same side one week after the first-stage surgery. The bilateral L3, L2, and L1 nerve roots and dural sacs were decompressed through the second-stage procedure (Figure 3). Bilateral nerve roots were exposed at all segments to ensure adequate lateral decompression of the lumbar canal (**Supplementary Video 1**).

**Figure 4:**
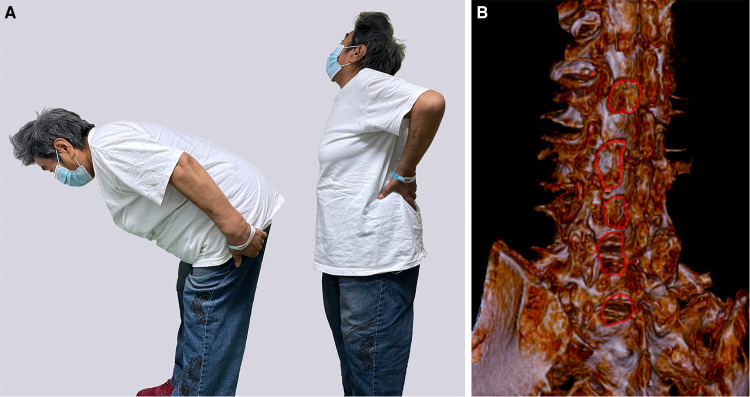
The patient achieved favorable flexion and extension of the back with little pain (**A**). Regions of bone resection after the two-stage procedure (red circles) (**B**).

**Figure 5:**
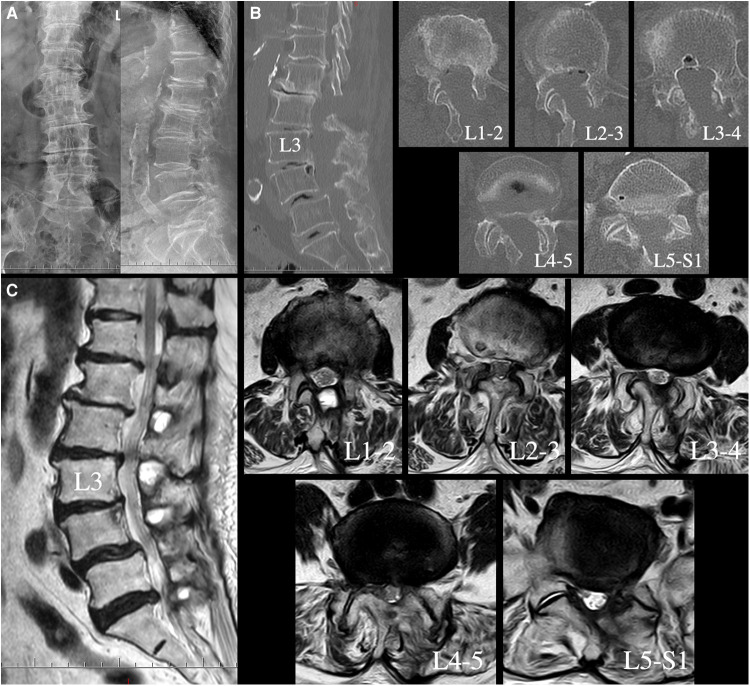
The lumbar X-ray radiograph of 1-year follow-up demonstrating all surgical segments remained stable (**A**). Lumbar CT of 1-year follow-up showing the bony structure changes of surgical segments (**B**). Sagittal and axial T2-weighted lumbar MRI revealing satisfactory decompression of lumbar canal from L1-2 to L5-S1 (**C**).

Celecoxib was given to relieve the low back pain postoperatively. The patient recovered to ambulate with a Boston brace after the second-stage surgery and rehabilitative training. The patient was discharged home after her low back pain was relieved, and she was able to bend and stretch her back (Figure 4A). Postoperative lumbar radiographs showed the range of laminectomies and decompressed lumbar spinal canals (Figure 4B). The patient has been followed-up for 1 year since the second-stage operation and reports significantly improved pain levels and the ability to complete daily activities. The 1-year follow-up's imagings showed satisfactory decompression of lumbar spinal canals, and the spondylolisthesis of L4 still not progressed (Figure 5).

## Discussion

LSS is caused by gradual degenerative narrowing of the spinal canal. According to a randomized controlled trial study, compared with decompression plus fusion surgery, single decompression surgery showed considerable clinical results (3). The ULBD technique was first reported by Young in 1988, and it has been rapidly improved by the use of various minimally invasive techniques, such as microscopy and microendoscopy. Nevertheless, the air medium required under microscopy and microendoscopy cannot provide a clear visual field, especially in contralateral decompression procedures (4, 5). Full endoscopic ULBD can achieve effective bilateral decompression *via* water medium, and several studies have reported favorable outcomes from this version of the procedure in the treatment of LSS (6). However, full endoscopic ULBD has a steep learning curve and a high rate of complications (7). ULBD *via* UBE is a relatively newly emerging technique that provides surgeons an alternative for conducting ULBD in a minimally invasive manner. Following the first report of this procedure from Egyptian and South Korean researchers, UBE-ULBD has been suggested to be a safe and effective surgery for LSS decompression (8, 9). Nevertheless, few studies have reported the clinical outcomes of multilevel UBE-ULBD, and its efficacy and safety remain unclear. In this case, a patient with multilevel LSS underwent five-level UBE-ULBD in two stages, which is the first report to our knowledge on such a large number of ULBD procedures for one patient.

For this patient, the long segmental lumbar fusion defects, including extensive detachment of the paravertebral extensors and limited back movement, were the reasons why we chose this minimally invasive, nonfusion surgery. In addition, this patient had no obvious degenerative spinal deformity or serious back pain. All her symptoms had developed as a result of compression of nerve roots and cauda equina. Staging the procedures can reduce the duration of each process, which is beneficial for the postoperative recovery of elderly patients. The range of bone resection in conventional ULBD mainly involves the partial unilateral lamina and internal cortex of the contralateral lamina. In this patient, bone resection involved the ventral side of the superior articular process due to decompression of the nerve root in the lateral recess. This procedure is also widely used in the full endoscopic version of ULBD surgery (10). Our experience with this patient demonstrates that ULBD with partial facet resection minimally damages the stability of the surgical segment, and the impairment of the paravertebral muscles was relatively limited. Additionally, the patient did not complain of obvious back pain during lumbar movement.

LSS is a very common pathological condition in elderly individuals. Complicating matters is that this pathological process frequently involves two or more levels, requiring the surgeon to attempt to balance wide-range decompression and spinal stability. UBE-ULBD could provide surgeons with a good alternative to expanded laminectomy or long segmental fusion. This minimally invasive procedure has remarkable advantages in producing early ambulation, inducing less incision pain, and requiring shorter hospital stays. All these factors could reduce the risk of postoperative complications, mortality and utilization in elderly patients. Moreover, many elderly patients who have multiple comorbidities, such as hypertension, diabetes mellitus and coronary heart disease, may have more opportunities to undergo lumbar surgery with the continuing development of minimally invasive nonfusion techniques.

UBE is an emerging minimally invasive spinal technique that can be performed for a variety of lumbar degenerative diseases, including multilevel lumbar spinal stenosis. ULBD *via* UBE can achieve safe and effective decompression, which may be crucial for allowing elderly patients to complete their daily activities. We presented a case of a patient who developed multilevel LSS and underwent two-stage, five-level UBE-ULBD, achieving a favorable clinical result.

## Data Availability

The original contributions presented in the study are included in the article/Supplementary Material, further inquiries can be directed to the corresponding author/s.

## Supplementary Material

**Supplementary Video 1** | The residual left lamina of L4 under the second-stage endoscopic visualization.
